# Porcelain bladder with ascending ureteral calcifications and seminal vesicle calcifications: CT imaging features of advanced urogenital schistosomiasis

**DOI:** 10.1016/j.radcr.2026.04.068

**Published:** 2026-06-02

**Authors:** Ismaila Mbodji, Ibrahima Niang, Mamadou Abass Diallo, Ahmadou Sow, Sokhna Ba

**Affiliations:** a Radiology Department, Fann University Hospital Center, Dakar, Senegal; b Faculty of Medicine, Pharmacy, and Dentistry, Cheikh Anta Diop University, Dakar, Senegal

**Keywords:** Urogenital schistosomiasis, Porcelain bladder, Ureteral calcification, Computed tomography

## Abstract

Urogenital schistosomiasis remains endemic in many regions of Africa and is a well-recognized cause of chronic lower urinary tract pathology. Longstanding infection may lead to bladder wall fibrosis, calcification, ureteral strictures, and hydronephrosis. We report the case of a middle-aged man presenting with chronic lower urinary tract symptoms and intermittent hematuria. Non-contrast computed tomography demonstrated circumferential bladder wall calcification consistent with a porcelain bladder, associated with bilateral ureteral mural calcifications and right-sided ureteropyelocaliceal dilatation. No renal parenchymal abnormalities were identified. This ascending pattern of urinary tract involvement, extending from the bladder toward the ureters with renal sparing, represents a characteristic imaging feature of chronic urogenital schistosomiasis and helps differentiate it from genitourinary tuberculosis, which typically originates in the kidney and progresses downward. Recognition of this imaging distribution is important for accurate diagnosis in endemic regions.

## Introduction

Urogenital schistosomiasis is a parasitic disease caused by *Schistosoma haematobium* and remains endemic in many parts of Africa and the Middle East [1]. Chronic infection results from deposition of parasite eggs within the bladder wall, inducing granulomatous inflammation, fibrosis, and dystrophic calcifications [1,2]. Longstanding disease may extend proximally along the urinary tract, leading to ureteral calcifications, strictures, and hydronephrosis [3]. Imaging plays a crucial role in identifying these complications and in differentiating schistosomiasis from other chronic inflammatory diseases of the urinary tract [2,4]. We report this case to illustrate the characteristic CT imaging pattern of advanced urogenital schistosomiasis and to emphasize the importance of recognizing its ascending pattern of urinary tract involvement for differentiation from genitourinary tuberculosis.

## Case presentation

A middle-aged man living in a riverine endemic region presented with chronic lower urinary tract symptoms, including dysuria, urinary frequency, and intermittent hematuria spanning several years. The patient reported repeated freshwater exposure since childhood. There was no history of previous urological surgery, known tuberculosis, or prior antiparasitic treatment.

Laboratory investigations revealed microscopic hematuria on urinalysis. Renal function was mildly impaired (serum creatinine 1.4 mg/dL). Urine cytology was negative for malignant cells. Parasitological confirmation by urine filtration for Schistosoma haematobium ova was not available at presentation. Chest radiography was unremarkable, with no features to suggest pulmonary tuberculosis.

Non-contrast computed tomography (CT) of the abdomen and pelvis was performed to evaluate the urinary tract. The examination demonstrated circumferential calcification of the bladder wall consistent with a porcelain bladder (Fig. 1). Calcifications of the seminal vesicles were also observed. Sagittal reconstructions revealed bilateral ureteral mural calcifications extending proximally (Figs. 2A and B). Associated right-sided ureteropyelocaliceal dilatation was present (Fig. 2A). No renal parenchymal calcifications, cortical scarring, or calyceal deformities were identified (Fig. 3).

**Fig. 1:**
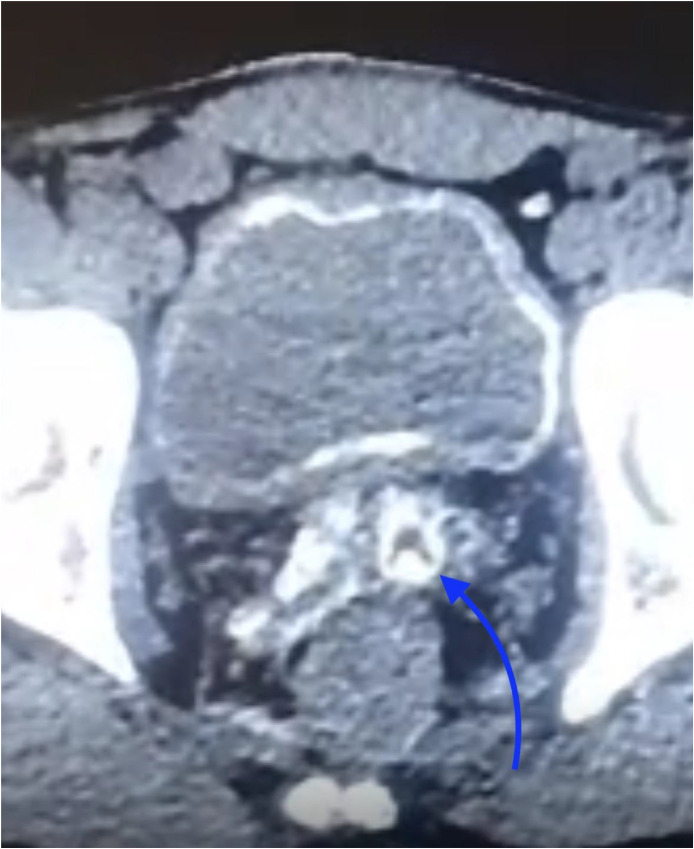
Axial non-contrast CT image demonstrating circumferential calcification of the bladder wall consistent with a “porcelain bladder.” Calcifications of the seminal vesicles are also visible (arrow).

**Fig. 2:**
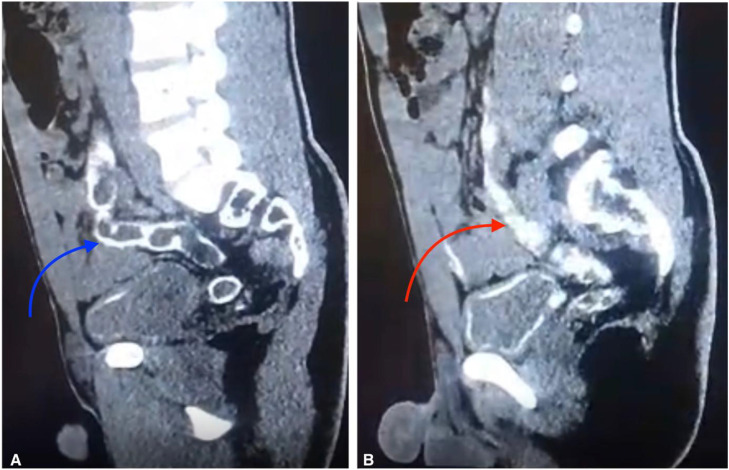
Sagittal CT reconstructions (right A; left B) demonstrating bilateral ureteral mural calcifications. Secondary right-sided ureteropyelocaliceal dilatation is visible on the right side (arrow).

**Fig. 3:**
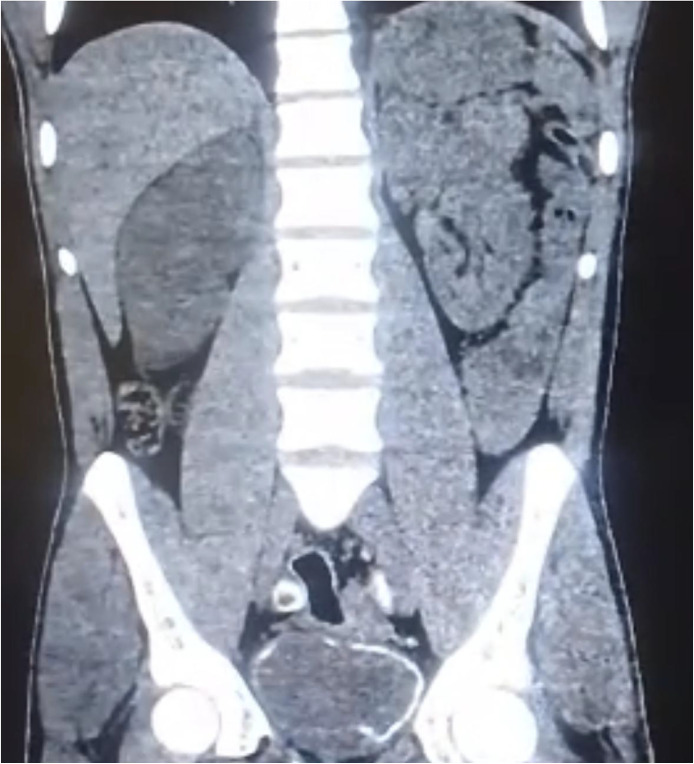
Coronal CT image confirming the absence of renal parenchymal abnormalities, cortical scarring, or calyceal deformities.

The combination of extensive bladder wall calcification, ascending ureteral involvement, and preserved renal morphology suggested advanced chronic urogenital schistosomiasis.

## Discussion

Urogenital schistosomiasis results from deposition of Schistosoma haematobium eggs in the bladder wall, producing chronic granulomatous inflammation that may progress to fibrosis and dystrophic calcification [1,2]. Bladder wall calcification is a well-recognized imaging manifestation of longstanding infection [2,5].

Ultrasound is an important complementary modality in the evaluation of urogenital schistosomiasis, particularly in endemic, resource-limited settings where CT may not be readily available. Sonographic findings include bladder wall thickening and echogenic mural deposits, ureteral dilatation, and upper urinary tract changes. In this case, ultrasound was not performed prior to CT. The role of ultrasound in staging and follow-up of urogenital schistosomiasis has been described in the WHO-standardized protocol [6].

In advanced disease, inflammatory changes may extend proximally along the ureters, producing mural calcifications and distal ureteral strictures [3]. These strictures may result in secondary upstream dilatation of the collecting system, as illustrated in our case by right-sided ureteropyelocaliceal dilatation.

An important imaging feature is the ascending distribution of urinary tract involvement. This pattern reflects extension of disease from the bladder toward the ureters and contrasts with genitourinary tuberculosis, which typically results from hematogenous renal infection followed by descending spread to the ureters and bladder [4,7]. Renal cortical scarring, cavitation, and parenchymal calcifications are common findings in renal tuberculosis but were absent in this case.

Chronic urogenital schistosomiasis is also associated with long-term complications including ureteral obstruction and an increased risk of squamous cell carcinoma of the bladder [8]. Recognition of this characteristic imaging distribution is therefore important for radiologists and clinicians practicing in endemic regions.

While schistosomiasis is the most common cause of bladder wall calcification in endemic regions, the differential diagnosis also includes genitourinary tuberculosis, transitional cell carcinoma with dystrophic calcification, and radiation cystitis [4,5,7,9]. Bladder calcification in tuberculosis is typically focal or segmental rather than circumferential and arises in the context of descending infection from the upper urinary tract. Malignant calcification is usually irregular and associated with a soft tissue mass. Radiation cystitis may cause bladder wall changes but calcification is uncommon. The circumferential “eggshell” pattern of bladder wall calcification, as seen in this case, is particularly characteristic of schistosomiasis [2,5]. Table 1 summarizes key imaging features that help differentiate chronic urogenital schistosomiasis from genitourinary tuberculosis.

**Table 1:** Imaging features differentiating urogenital schistosomiasis from genitourinary tuberculosis.

Imaging feature	Urogenital schistosomiasis	Genitourinary tuberculosis
Direction of spread	Ascending (bladder → ureters → kidneys)	Descending (kidneys → ureters → bladder)
Bladder calcification	Circumferential ***(“eggshell”/“porcelain”)***	Focal/irregular; small contracted bladder ***(“thimble bladder”)***
Ureteral involvement	Bilateral mural calcifications; distal ureteral strictures	Ureteral strictures; pipe-stem calcification; irregular caliber
Predominant site of calcification	Bladder wall	Renal parenchyma/collecting system
Renal parenchyma	Typically spared in early-to-advanced disease	Cortical scarring, cavitation, parenchymal calcifications ***(“putty kidney”)***
Other findings	Seminal vesicle calcifications; increased bladder cancer risk	Adrenal or genital TB; pulmonary involvement

From an imaging perspective, the combination of circumferential bladder wall calcification (“porcelain bladder”), ascending ureteral mural calcifications, and relative preservation of renal parenchyma strongly suggests chronic urogenital schistosomiasis [1,2,3].

## Conclusion

Porcelain bladder associated with ascending ureteral calcifications and preserved renal morphology represents a classic imaging pattern of advanced chronic urogenital schistosomiasis and provides an important clue for distinguishing this condition from genitourinary tuberculosis. Recognition of this characteristic distribution on CT may facilitate accurate diagnosis and appropriate clinical management in endemic regions.

## Author contributions

**Ismaila Mbodji:** data acquisition, image collection, and initial drafting of the manuscript. **Ibrahima Niang:** conception of the study, supervision of imaging interpretation, and critical revision of the manuscript. **Mamadou Abass Diallo and Ahmadou Sow:** clinical data collection and participation in manuscript drafting. **Sokhna Ba:** supervision of the work and final approval of the manuscript. All authors read and approved the final version of the manuscript.

## Declaration of generative AI and AI-assisted technologies in the writing process

During the preparation of this work the authors used Claude AI (sonnet 4.6) for language editing in order to *improve readability*. After using this tool, the author reviewed and edited the content as needed and take full responsibility for the content of the published article.

## Patient consent

Written informed consent for publication of this case report and associated images was obtained from the patient.
